# Metabarcoding analysis of oral microbiome during pregnancy

**DOI:** 10.3389/fcimb.2024.1477703

**Published:** 2024-12-17

**Authors:** Fatiha M. Benslimane, Layla I. Mohammed, Haya Abu-Hijleh, Sara Suleiman, Sonia Boughattas, Zain Zaki Zakaria, Eleni Fthenou, Maha Al-Asmakh

**Affiliations:** ^1^ Biomedical Research Center, Qatar University, Doha, Qatar; ^2^ Department of Biomedical Sciences, College of Health Sciences, Qatar University (QU) Health, Qatar University, Doha, Qatar; ^3^ Vice President for Medical and Health Sciences Office, QU Health, Qatar University, Doha, Qatar; ^4^ Qatar Biobank for Medical Research, Qatar Foundation, Doha, Qatar

**Keywords:** pregnancy, oral microbiome, gut microbiota, salivary microbiome, Qatari birth cohort (QbiC), oxford nanopore sequencing

## Abstract

Pregnancy is a dynamic physiological process involving significant hormonal, immune, and metabolic changes to support fetal growth and development. This study investigates the changes in salivary microbiome and biochemical markers from the second to the third trimester of pregnancy. Saliva samples were collected from 45 pregnant women enrolled in the Qatar Birth Cohort study at two time points (second and third trimesters). DNA was extracted and subjected to 16S rRNA gene sequencing using Oxford Nanopore Technology. Microbial diversity and taxonomic analyses were performed, along with correlation analyses between microbial abundance and clinical parameters. Biochemically, significant increases in BMI, pulse rate, HbA1c, LDL, total cholesterol, and triglycerides were observed in the third trimester compared to the second. Microbial diversity analysis revealed significant changes in microbial richness and composition. Taxonomy analysis showed a significant 3-fold increase in Bacteroidota. Also, a significant decline in *Selenomonas* and a significant increase in *Veillonella*, specifically *Veillonella dispar* and *Veillonella atypica*, as well as an increase in *Granulicatella* were observed in the third trimester, along with a significant decrease in *Streptococcus sanguinis*. Correlation analysis during the second trimester revealed positive associations between BMI, cholesterol, LDL, and *Selenomonas*, and negative correlations with *Streptococcus* and *Gemella*. In the third trimester, BMI was negatively correlated with *Campylobacter*, glucose levels were negatively correlated with *Neisseria*, and triglyceride levels were negatively correlated with *Prevotella*. These findings highlight significant biochemical and microbial shifts during pregnancy, underscoring the importance of monitoring oral health and metabolic changes in pregnant women.

## Introduction

Pregnancy represents a remarkable transformation for a woman’s body. A cascade of hormonal, immune, and metabolic changes orchestrate the nurturing and development of a healthy fetus (Murray and Hendley, 2020). In recent years, scientific curiosity has turned towards the fascinating role of the human microbiome during this critical period (Fasano and Flaherty, 2021).

The gut microbiome, long recognized for its influence on digestion and overall health, exhibits dramatic shifts throughout pregnancy (Yao et al., 2021). Research suggests a rise in specific bacterial groups like Proteobacteria and Actinobacteria, while others, particularly butyrate-producing bacteria, see a decline (Fu et al., 2019). These alterations are believed to be adaptations that support the body’s heightened metabolic demands during pregnancy, ultimately contributing to fetal growth (De Siena et al., 2021). Additionally, the gut microbiome might play a role in regulating weight gain through mechanisms like nutrient absorption and immune system stimulation (Yoo et al., 2020).

The oral cavity harbors another complex and diverse microbial ecosystem – the oral microbiome – which is also thought to be susceptible to the hormonal and immunological fluctuations that occur during pregnancy (Sedghi et al., 2021). Studies have shown an increase in total bacterial counts in pregnant women, including some bacteria associated with gum disease (Jang et al., 2021; Saadaoui et al., 2021). Interestingly, correlations have been observed between oral infections and pregnancy complications, suggesting a potential link between the health of the oral microbiome and the course of pregnancy (Wen et al., 2023). Furthermore, a mother’s oral microbiome may influence the development of her infant’s oral microbiome during the perinatal period, potentially impacting their future oral and systemic health (Nardi et al., 2021).

This complex community of microorganisms residing in various body sites, including the gut and oral cavity, undergoes distinct alterations throughout gestation (Sedghi et al., 2021; Mohammed et al., 2024). Investigating these microbiome fluctuations across trimesters holds promise for elucidating potential associations with maternal health and pregnancy outcomes (Baud et al., 2023). Therefore, this study aims to examine changes in the salivary microbiome composition during pregnancy across the second and third trimesters. By leveraging the Qatari Birth Cohort (QbiC) we aim to explore the dynamic shifts in the oral microbiomes and illuminate the intricate interplay between the oral microbiome and the course of pregnancy.

## Methodology

### Sample collection and participant criteria

The proposed study was designed to gather saliva samples from pregnant women enrolled in the Qatar Birth Cohort study at Qatar Biobank (QBB). These samples were obtained from 45 pregnant women taken at two different time points (second and third trimesters), making a total of 90 samples selected from the QBB repository. Pregnant women who have lived in Doha for at least 15 years and were anticipated to deliver their babies in Qatar were eligible to participate in this study. Participants had to be residents in the study area, aged over 18 years, without any communication handicaps. Women with clinically diagnosed metabolic, metastatic, or chronic infectious diseases were excluded from the study. Additionally, relevant information on physical activity, feeding habits, socio-economic status, smoking habits, disease history, medication, and family history were obtained from the Qatar Biobank.

This study was conducted in accordance with the ethical principles outlined in the Declaration of Helsinki. Approval for the study was obtained from the Institutional Review Board (IRB) of QBB, with the ethical approval reference number [QF-QBB-QBIC-RES-ACC-0225-0129]. All participants provided written informed consent before their inclusion in the study. Confidentiality of the participants’ data was strictly maintained throughout the research process, and the samples were anonymized to ensure privacy. The study adhered to all relevant national and international regulations and guidelines for research involving human subjects.

### DNA extraction

Saliva samples collected from pregnant women were retrieved from the QBB repository and transported on ice from to Qatar University. Genomic DNA extraction of the samples was achieved using the DNeasy Blood & Tissue Kits (Qiagen^®^, Germany) according to the manufacturer guidelines.

### Library preparation and sequencing

The extracted DNA from the different saliva specimens were subjected to purification and quantification assessment using the Nanodrop ratio A260/A280 and the Qubit dsDNA High Sensitivity Assay kit, respectively. A total of 10ng of pure DNA was utilized for the library preparation using ONT’s 16S Barcoding Kit (SQK-16S024) according to the manufacturer’s guidelines. Briefly, specimen genomic DNA was subjected to barcoding and amplification of the full length of 16S rRNA gene using LongAmp Hot Start Taq 2X Master Mix (New England Biolabs), and the primer set 27F/1492R containing 5’ tags to facilitate the ulterior ligase-free attachment of the Sequencing Adapters. The PCR end products were then purified using CleanMag^®^ Magnetic Beads (Paragon Genomics) at a 0.6X ratio. All barcoded libraries were pooled in Elution Buffer, pH8, at equal ratios of 60 fmoles. The Rapid Sequencing Adapter (RAP) was then incorporated into the pooled libraries and incubated for 5 min at room temperature before adding the Sequencing Buffer (SQB) and the Loading Beads (LB) followed by loading a primed Flongle Flow Cell R9.4.1 (FLO-FLG001). The sequencing was launched under high accuracy base calling parameter and with a QScore >7. Base calling was performed using MinKNOW software (3.6.5).

### Bioinformatics & statistical analysis

Fastq_pass files were analyzed using EPI2ME software (V5.1.3) using the 16S workflow (wf-16S, V0.0.3) and minimap2 aligner (2.26-r1175) under the default conditions: reads size ranging from 1200bp to 1700bp using a Blast E-value filter of [e=0.01] and showing the minimum coverage was 80% and 80% identity. The resolution was set to the species level. Phylum, genus and species read csv files were downloaded from EPI2ME and the relative abundance was calculated. The relative abundance was filtered to be ≥ 5%. R programming language version 4.3.1 (2023) was utilized for filtration and statistical analysis. The alpha diversity indices (sobs and Shannon) and beta diversity (Bray-Curtis dissimilarity) were computed using vegan package 2.6.4 (Oksanen et al., 2015). Significance tests for clinical were conducted using either a paired t-test or paired Wilxcon test, depending on the data distribution. Microbial significance was assessed using a paired t-test. A p-value less than 0.05 was considered statistically significant. The Spearman correlation test was performed to indicate correlation between the microbial taxa at the genus level and clinical tests. Plots were generated using ggplot2 package version 3.4.3 (Wickham, 2016) and indicating the significance ggsignif package was used version 3.4.3 (Ahlmann-Eltze and Patil, 2021).

## Results

### Characteristics of the study population

Demographic data of the 45 pregnant women was obtained from Qatar Biobank in their second and third trimesters (**Table 1**). After excluding individuals with missing information, the study included 35 females, among whom 14% were Qatari. The mean age of the participants was 29 ± 5.12 years. Approximately 31% of the participants were experiencing their first pregnancy, while about 68% had multiple births, and 7% had experienced a previous miscarriage. Participants who took painkillers, antipyretics, or antibiotics accounted for 51.43% of the total, and all were included in the analysis.

**Table 1:** Demographic Characteristics and Reproductive Outcomes of the Study Population.

Characteristic	Value
Qatari (%)	14.29
Non-Qatari (%)	85.71
Age (years)*	28.86 ± 5.12
First Born (%)	31.43
Multiple births (%)	68.57
Miscarriage or Abortion (%)	7

*Numbers are presented as percentage or mean ± standard error.

### Clinical changes during second and third pregnancy trimesters

The body mass index (BMI) and pulse rate showed a significant increase in the third trimester compared to the second trimester (p< 0.001), **Table 2**. Due to missing clinical data from either the second or third trimester in four individuals, these participants were excluded from the study. Consequently, a total of 31 participants were included in the analysis of clinical characteristics. There was a significant increase in HbA1c and LDL levels, p value of 0.047 and 0.005, respectively. Additionally, there was a more profound significant increase in total cholesterol (1.25 fold) and triglycerides (1.75 fold), **Table 2**.

**Table 2:** Clinical characteristics during the second and third pregnancy trimesters.

Parameters	Second Trimester	Third Trimester	P. Value
**BMI**	28.16 ± 4.96	31.09 ± 4.83	**<0.001**
**Systolic blood pressure**	98.23 ± 8.36	98.17 ± 7.49	0.954
**Diastolic blood pressure**	54.86 ± 5.71	55.57 ± 5.83	0.500
**Pulse Rate (BPM)**	78.43 ± 9.89	86.23 ± 12.44	**<0.001**
**Cholesterol Total (mmol/L)**	4.78 ± 0.84	5.97 ± 0.95	**<0.001**
**Ferritin (ng/ml)**	45.29 ± 87	73.26 ± 183.4	0.281
**Folate (nmol/L)**	38.98 ± 18.4	32.16 ± 19.7	0.072
**Glucose (mmol/L)**	4.24 ± 0.61	4.41 ± 0.69	0.227
**HbA1C (%)**	4.99 ± 0.27	5.07 ± 0.31	**0.047**
**HDL (mmol/L)**	1.77 ± 0.38	1.87 ± 0.43	0.063
**Iron (µmol/L)**	14.17 ± 6.5	19.77 ± 20.27	0.592
**LDL (mmol/L)**	2.38 ± 0.8	2.91 ± 1.09	**0.005**
**Triglyceride (mmol/L)**	1.41 ± 0.47	2.42 ± 0.72	**<0.001**

HbA1C: Hemoglobin A1C, HDL: High density lipoprotein, LDL: Low density lipoprotein. Numbers are presented as mean ± standard error. BMI to pulse rate is average of 35 participants while the rest clinical parameters are average of 31 participants. The bold values indicate significance.

### Microbial diversity

**Figure 1:**
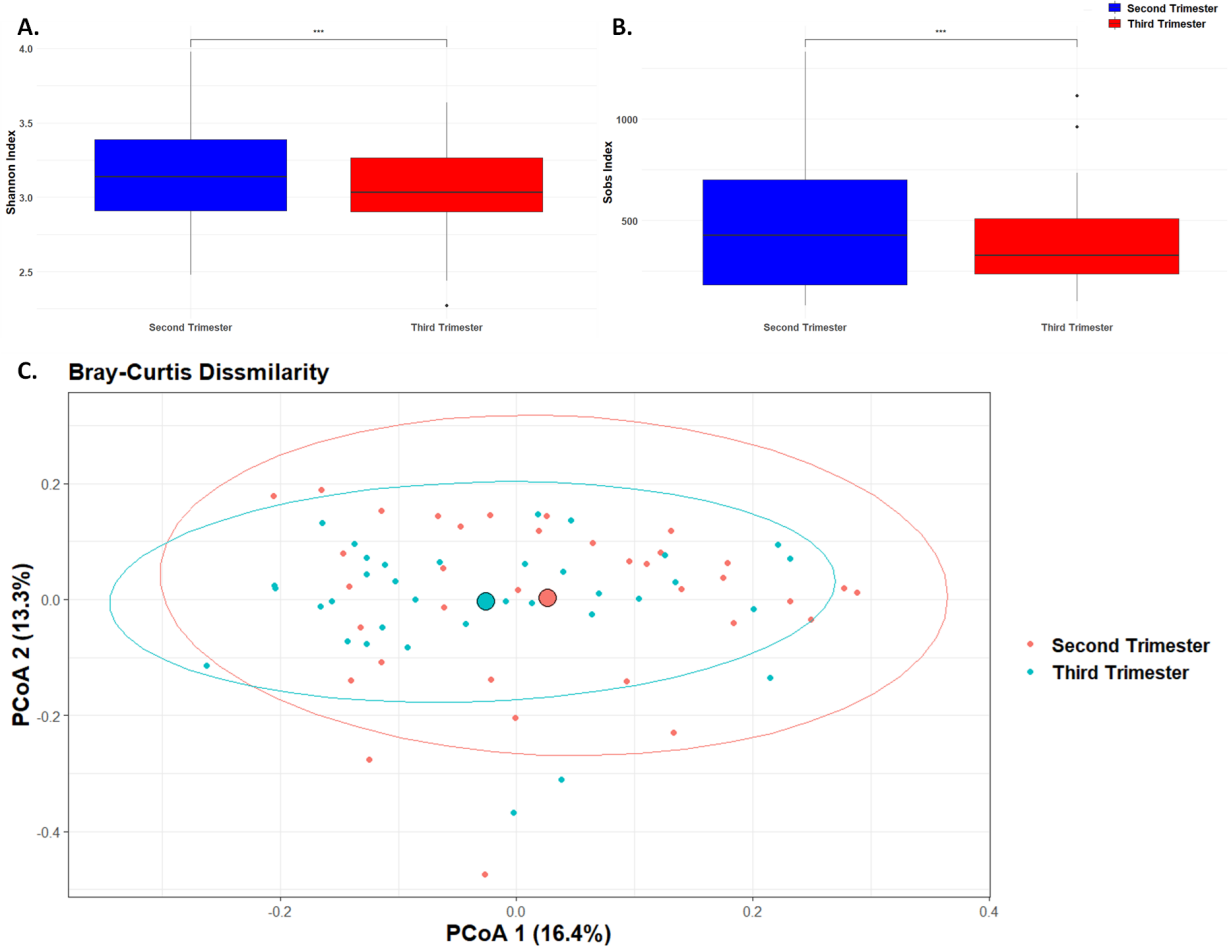
Microbial Diversity. Box plot of differences in observed species richness and evenness using **(A)**. Shannon diversity index and **(B)**. Sobs value. (*** p< 0.001). **(C)**. Principal component analysis plot representing beta diversity distance matrices of the Bray–Curtis distance comparing the sample distribution between the second and third trimesters. The red dots represent samples from the second trimester, and the blue dots represent samples from the third trimester.

Alpha diversity, which measures the richness and evenness of microbial species within a sample, showed significant changes from the second to the third trimester of pregnancy. Specifically, the observed species (Sobs, **Figure 1B**) decreased from 499.6 ± 343.3 in the second trimester to 388.0 ± 222.7 in the third trimester, with a highly significant p-value of less than 0.0001. Similarly, the Shannon diversity index (**Figure 1A**), which accounts for both abundance and evenness of the species present, also showed a significant decrease from 3.15 ± 0.34 in the second trimester to 3.05 ± 0.31 in the third trimester (p < 0.0001). This reduction in the Shannon diversity index indicates a decrease in both the number of species and their evenness during later stages of pregnancy, possibly indicating a shift towards a less complex microbial community as pregnancy progresses.

Beta diversity, which measures the differences in microbial community composition between samples, was assessed using the Bray-Curtis dissimilarity index (**Figure 1C**). The Bray-Curtis dissimilarity index indicated a significant shift in the overall microbial community composition between the second and third trimesters (p < 0.01). This indicates significant changes in the overall composition and structure of the microbial community as pregnancy progresses, likely reflecting shifts in species abundance or presence.

### Taxonomy analysis

**Figure 2:**
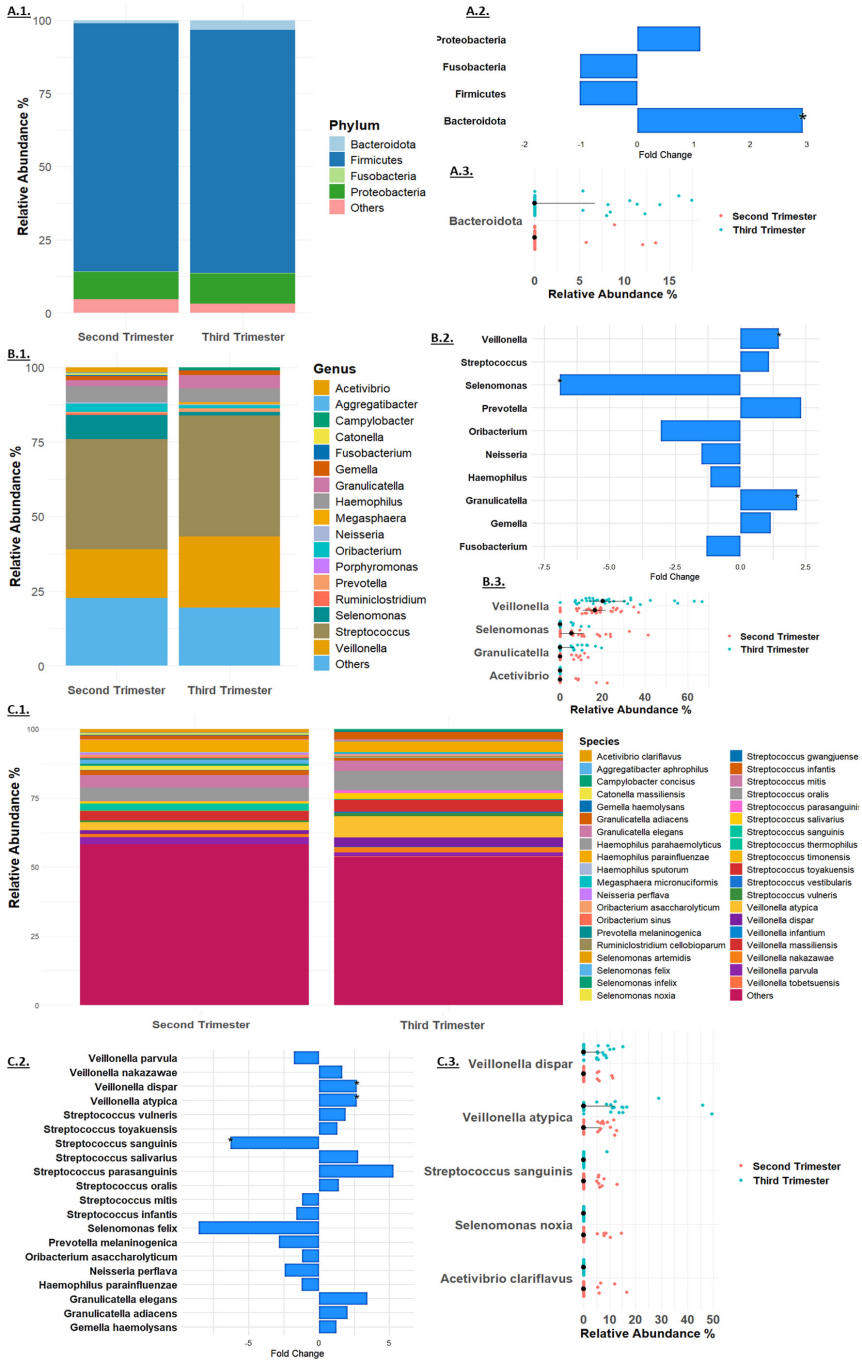
Taxonomy Analysis at the **(A)**. Phylum, **(B)**. Genus and **(C)**. Species levels. Microbial relative mean abundance at the **(A.1)**. Phylum, **(B.1)**. Genus level and **(C.1)** Species levels. Relative fold change of taxa **(A.2)**. Phylum, **(B.2)**. Genus and **(C.2)**. Species levels. Taxa that exhibited significant change at the **(A.3)**. Phylum, **(B.3)**. Genus and **(C.3)**. Species levels in each sample in the second trimester (red dots) and third trimester (blue dots).

The 16S rRNA gene sequencing analysis at the phylum level detected a total of four phyla: Firmicutes, Proteobacteria, Bacteroidota, and Fusobacteria (**Figure 2A**). Among these, Firmicutes was the most abundant, with a relative abundance of 84.69% in the second trimester and 82.98% in the third trimester (**Figure 2A.1**). There were minimal changes in the relative abundance of most bacterial phyla between the second and third trimesters. Firmicutes showed a slight, non-significant decrease with a fold change of -1.02 (p = 0.54), while Proteobacteria exhibited a minor, non-significant increase from 9.27% to 10.28%, corresponding to a fold change of 1.11 (p = 0.71). Fusobacteria remained relatively stable, with a minimal change from 0.26% to 0.25% and a fold change of -1.01 (p = 0.99, **Figure 2A.2**). In contrast, Bacteroidota displayed a significant increase in relative abundance, rising from 1.15% in the second trimester to 3.34% in the third trimester, with a fold change of 2.92 (p = 0.03, **Figures 2A.2, A.3**). These findings suggest that while most bacterial phyla remained stable, Bacteroidota underwent a significant shift during the course of pregnancy.

At the genus level (**Figure 2B**), *Streptococcus* was the most abundant, increasing slightly from 37.01% to 40.46% (fold change 1.09, p = 0.35, **Figure 2B.1**). Significant changes were observed in other genera: *Granulicatella* increased significantly from 2.05% to 4.47% (fold change 2.18, p = 0.03), and *Veillonella* rose from 16.19% to 23.87% (fold change 1.47, p = 0.013). *Selenomonas* showed a significant decrease from 8.03% to 1.16% (fold change -6.90, p = 0.0014), while *Acetivibrio* exhibited a notable reduction to non-detectable levels in the third trimester (p = 0.04, **Figures 2B.2, B.3**). Other genera, such as *Gemella*, *Haemophilus*, *Prevotella*, and *Fusobacterium*, showed minor changes, and several genera including *Ruminiclostridium*, *Oribacterium*, *Catonella*, *Aggregatibacter*, *Porphyromonas*, *Megasphaera*, and *Campylobacter* were non-detectable in the third trimester.

At the species level (**Figure 2C**), *Streptococcus mitis* was the most abundant, with a relative abundance of 4.52% in the second trimester and 3.80% in the third trimester (fold change -1.19, p = 0.51, **Figure 2C.1**). Significant changes were observed in several other species: *Granulicatella adiacens* increased from 1.36% to 2.79% (fold change 2.05, p = 0.07), and *Veillonella dispar* rose from 1.26% to 3.38% (fold change 2.68, p = 0.04). *Veillonella atypica* also increased significantly from 2.88% to 7.69% (fold change 2.67, p = 0.03). *Streptococcus sanguinis* showed a significant decrease from 1.61% to 0.26% (fold change -6.28, p = 0.04, **Figures 2C.2, C.3**). Several species exhibited reductions to non-detectable levels in the third trimester, including *Selenomonas noxia* (p = 0.02), *Selenomonas timonensis*, *Acetivibrio clariflavus* (p = 0.04), and others. These findings underscore the dynamic shifts in the microbiome composition during pregnancy, highlighting the significant changes at multiple taxonomic levels.

### Correlation analysis

**Figure 3:**
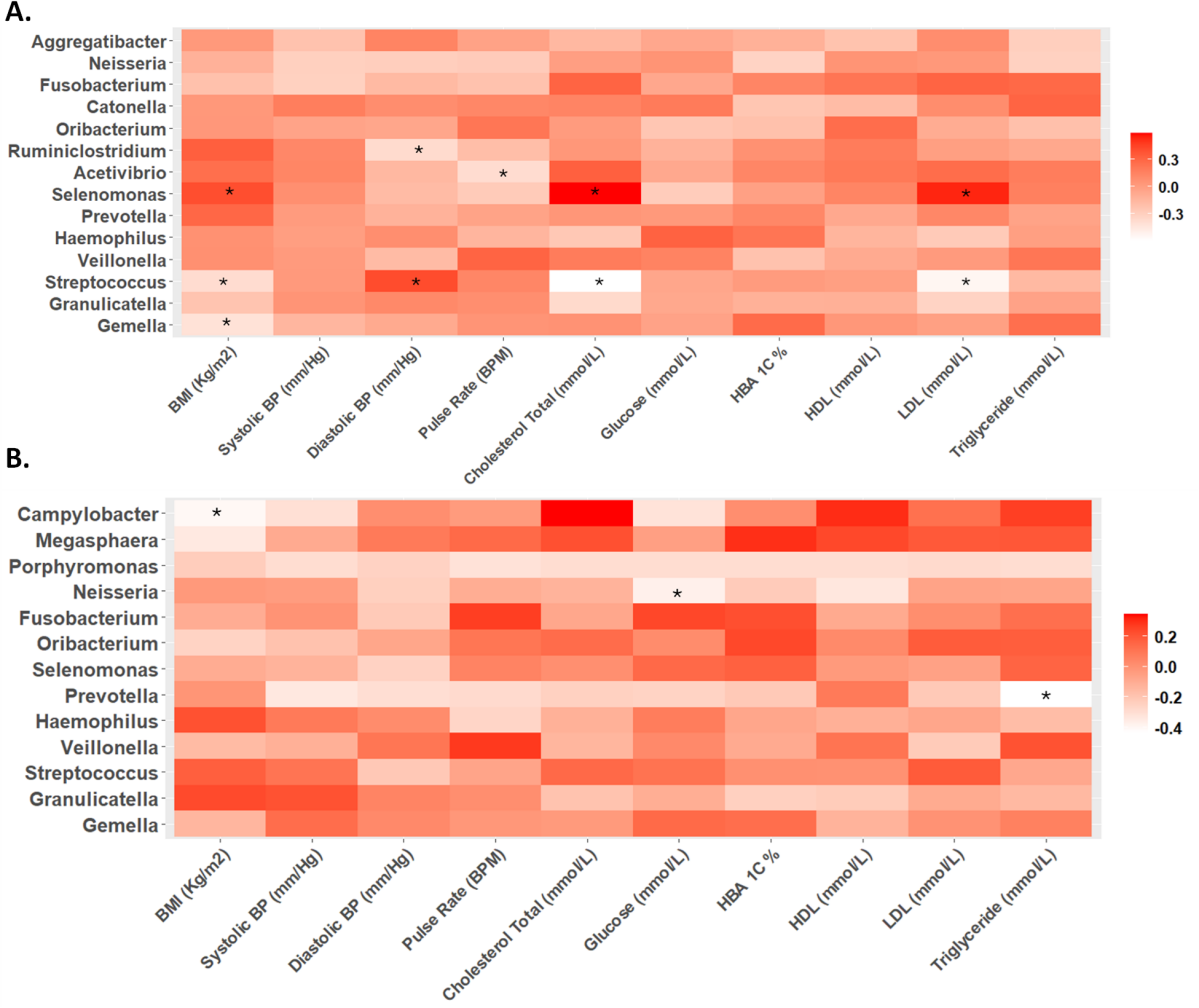
Heat maps of Spearman correlation analysis between relative abundance of microbes at genus level and biochemical parameters in the **(A)** second and **(B)** third trimester. The color intensity (white to red) indicates the r-value, with asterisks denoting significance (p-value < 0.05).

During the second trimester, significant correlations were observed between various physiological markers and microbial taxa. Specifically, BMI, cholesterol levels, and LDL were positively associated with *Selenomonas* abundance, while inversely correlated with *Streptococcus*. Additionally, BMI exhibited a negative correlation with *Gemella*. Diastolic blood pressure showed a positive correlation with *Selenomonas* abundance and a negative correlation with *Ruminiclostridium* (**Figure 3A**).

In contrast, during the third trimester, different correlation trends were observed. While the positive correlation between diastolic blood pressure and *Selenomonas*, as well as the negative correlation between diastolic blood pressure and *Ruminiclostridium*, remained, other significant correlations emerged. BMI showed a negative correlation with *Campylobacter*, and glucose levels were inversely correlated with *Neisseria*. Triglyceride levels displayed a negative correlation with *Prevotella* abundance (**Figure 3B**).

## Discussion

Pregnancy is a transformative period marked by numerous physiological changes, including fluctuations in hormone levels and immune responses (Nuriel-Ohayon et al., 2016). Emerging evidence has highlighted alterations in the oral microbiome during this time. The hormonal and immune modulations associated with pregnancy may significantly influence oral microbiota composition, with potential consequences for both maternal and fetal health (Mockridge and Maclennan, 2019; Saadaoui et al., 2021). Understanding these dynamic changes is crucial for enhancing maternal oral health and, by extension, broader systemic health during this critical period.

Our study demonstrated several significant shifts in both clinical and microbial parameters throughout pregnancy. The observed increase in pulse rate, a typical physiological response to pregnancy, aligns with known cardiovascular changes driven by elevated estrogen, progesterone, and prostaglandin levels (La et al., 2022). These hormones contribute to increased diastolic and stroke volumes, along with a higher heart rate, resulting in a progressive rise in cardiac output during the latter stages of pregnancy (Morton, 2021; La et al., 2022). This heightened cardiac output primarily serves the growing fetus and prepares the mother’s body for postpartum lactation (Zakaria et al., 2022).

Regarding lipid metabolism, pregnancy induces dynamic alterations that transition from an anabolic state in the first two trimesters to a catabolic phase in the third trimester, as insulin sensitivity decreases (Elkus and Popovich, 1992; Herrera, 2002; Buggio et al., 2019). Our study found significant increases in cholesterol, LDL, and triglycerides during pregnancy, consistent with the typical metabolic shifts of pregnancy (Herrera, 2002). These lipid changes are influenced by hormonal fluctuations and metabolic adaptations, which also have an impact on gut microbiota diversity and composition (Napso et al., 2018). Oral diseases such as periodontal disease and gingivitis are more likely during pregnancy due to increased sex steroid hormones and potential microbiome dysbiosis, emphasizing the importance of periodontal care (Boggess and Society for Maternal-Fetal Medicine Publications Committee, 2008; Silk et al., 2008; Balan et al., 2018; Figuero et al., 2020; Rapone et al., 2020; Giannella et al., 2023; Wild and Feingold, 2023).

The notable shifts in both alpha and beta diversity in our cohort suggest significant changes in microbial composition as pregnancy progresses. Specifically, the decline in alpha diversity in the third trimester likely reflects physiological adaptations to pregnancy, such as increased insulin resistance and weight gain (Nuriel-Ohayon et al., 2016; Zakaria et al., 2022). This reduction in diversity could be beneficial for fetal growth, illustrating how the maternal microbiome adapts to meet the needs of pregnancy.

Further, our findings on weight gain align with previous studies showing that changes in microbial diversity are associated with normal weight gain during pregnancy, especially in relation to the increased abundance of *Bacteroides* (Santacruz et al., 2010; Li et al., 2023; Sinha et al., 2023; Strobel et al., 2023). The increased presence of *Bacteroides*, a member of the *Bacteroidetes* phylum, has been linked to improved lipid profiles and folic acid levels, supporting the maternal metabolic adaptations required for a healthy pregnancy (Santacruz et al., 2010). This is consistent with previous research reporting a similar trend in the relative abundance of *Bacteroidetes* and *Firmicutes*, with *Bacteroides* showing a notable increase from the second to the third trimester (Gao et al., 2024). However, it is important to note that we did not observe any significant changes at the species level within the *Bacteroidota* phylum; nonetheless, the increase in the overall *Bacteroidota* abundance during pregnancy may still suggest broader functional roles within the maternal microbiome.

At the genus level, our observation of a decrease in the relative abundance of *Selenomonas* during the third trimester may be linked to hormonal changes and immune modulation, which are known to alter the oral microbiome (Balan et al., 2018; Xu et al., 2020; Giannella et al., 2023). Although literature on *Selenomonas* during pregnancy is sparse, previous studies suggest its abundance is associated with gestational diabetes mellitus (GDM) and other pregnancy complications (DiGiulio et al., 2015; Tuominen et al., 2018). The reduced abundance of *Selenomonas* in our study cohort could reflect positive oral health behaviors among participants, a factor previously linked to improved pregnancy outcomes (Silk et al., 2008).

Conversely, the increase in the mean relative abundance of *Veillonella* and *Granulicatella* during the third trimester is consistent with their roles as opportunistic pathogens, often associated with oral diseases such as gingivitis (Eribe and Olsen, 2017; Knapp et al., 2017; Yang et al., 2019). *Veillonella*, in particular, has been implicated in the translocation of bacteria from the oral cavity to the placenta in murine models, suggesting its potential involvement in maternal-fetal microbial transmission (Fardini et al., 2010; Gomez-Arango et al., 2017). Its increase in our study further supports findings that pregnancy promotes the growth of certain gram-negative anaerobic bacteria in the oral cavity due to hormonal changes (Beighton et al., 2008; Yokoyama et al., 2008; Gürsoy et al., 2009; MaChado et al., 2012; Bäckhed et al., 2015; Fujiwara et al., 2017; Massoni et al., 2019).

*Granulicatella*, another gram-positive bacterium, has been linked to various infections and metabolic disturbances, including dyslipidemia and obesity (Gardenier et al., 2011; Crusell et al., 2018; Wu et al., 2018; Sparvoli et al., 2020; Zhao et al., 2020; Aranaz et al., 2021; Dong et al., 2021; Li et al., 2022; Gao et al., 2024). The significant increase in its abundance during pregnancy, as observed in our study, may have implications for lipid metabolism, maternal inflammation, and potential complications such as gestational diabetes (Crusell et al., 2018). Our findings contribute to the growing body of research indicating that *Granulicatella* plays a role in the maternal microbiome and metabolic health during pregnancy.

*Streptococcus sanguinis*, a component of the core microbiome, exhibited higher abundance in the second trimester, suggesting that hormonal changes and dietary habits during pregnancy influence its prevalence (Santacroce et al., 2023). Additionally, correlations between specific bacterial genera and metabolic parameters indicate complex interplays during pregnancy. For instance, the negative correlation between *Streptococcus* levels and metabolic markers supports previous findings regarding probiotics and their role in improving lipid profiles and inflammatory markers during pregnancy (Asemi et al., 2012; Jafarnejad et al., 2016).

*Gemella*, linked to maternal health, showed a tendency to decrease as BMI increased, suggesting that maternal weight may influence microbial composition (Yao et al., 2021). The correlation between diastolic blood pressure and *Selenomonas* in the second trimester further illustrates the relationship between cardiovascular alterations and microbial dynamics (Fu et al., 2019). Studies indicate that elevated DBP is associated with adverse pregnancy outcomes, highlighting the need for further exploration of this correlation (Yoo et al., 2020; De Siena et al., 2021).

In the third trimester, negative correlations between BMI, glucose, and *Campylobacter*, *Neisseria*, and *Prevotella* suggest that metabolic changes influence oral microbiota composition (Zhang et al., 2010). Specifically, *Campylobacter* levels were associated with oral hygiene practices, indicating a link between oral health and microbial ecology (La et al., 2022). The negative correlation between *Neisseria* and glucose levels during the third trimester implies its potential role in glucose metabolism, which warrants further investigation (Morse Stephen et al., 1974; Exley Rachel et al., 2005; Exley Rachel et al., 2007; Crusell et al., 2020; Ren et al., 2023).

## Conclusion

In conclusion, our study reveals significant changes in both clinical and microbial parameters during pregnancy, underscoring the intricate relationship between maternal health and the microbiome. These findings enhance our understanding of how these changes may influence maternal and fetal health outcomes, emphasizing the need for vigilant clinical oversight throughout pregnancy. Recognizing these alterations is crucial for establishing appropriate benchmarks for maternal health. Additionally, while medication use, including antibiotics, was recorded, the lack of specific exclusion criteria for antibiotic use should be considered a limitation, as it may impact microbiome composition. Further research is warranted to explore the mechanisms underlying these changes and their potential long-term effects on both maternal and offspring health.

## Data Availability

The original contributions presented in the study are publicly available. This data can be found here: NCBI - PRJNA1195396.
